# Splenic Torsion in a Patient With Situs Inversus Totalis and Polysplenia: Challenging Diagnosis and Treatment of a Rare Case

**DOI:** 10.7759/cureus.102609

**Published:** 2026-01-29

**Authors:** Emilie Zhu, Héloïse Giron, Maurice Matter, Michael Schneider

**Affiliations:** 1 Visceral Surgery, Hôpital de Morges, Ensemble Hospitalier de la Côte (EHC), Morges, CHE; 2 Visceral Surgery, Hôpital Riviera-Chablais, Rennaz, CHE; 3 Visceral Surgery, Lausanne University Hospital, University of Lausanne, Lausanne, CHE; 4 Visceral Surgery, Lausanne University Hospital, Lausanne, CHE

**Keywords:** polysplenia, situs inversus, splenectomy, splenic ischemia, splenic torsion

## Abstract

Splenic torsion is a rare condition caused by the twisting of the splenic pedicle, potentially leading to parenchymal infarction. We report a case of splenic torsion in a young patient with situs inversus totalis, isolated polysplenia, and a wandering spleen, without other cardiothoracic or digestive anomalies. An 18-year-old female, already known for situs inversus totalis, presented in the emergency department of a regional hospital with sudden right abdominal pain associated with vomiting. Laboratory tests showed leukocytosis and elevated C-reactive protein. Abdominal CT confirmed the situs inversus totalis and revealed a polysplenia with a well-delimited hypodense mass suspected of infarction of the main spleen due to the torsion of its vascular pedicle. After the emergency patient’s transfer to our tertiary hospital (Lausanne University Hospital, Lausanne, CHE), hand-assisted laparoscopic splenectomy was performed. The postoperative course was uneventful, and the patient was discharged on the third postoperative day.

Splenic torsion is a rare but serious condition requiring prompt diagnosis and surgical intervention. Patients with situs inversus, polysplenia, or a wandering spleen are predisposed to splenic torsion due to abnormal anatomy. Imaging by CT is crucial for diagnosis, and laparoscopic splenectomy remains a safe and effective treatment when infarction occurs.

## Introduction

Splenic torsion is a rare condition defined by twisting of the splenic pedicle, potentially resulting in parenchyma infarction. A recent systematic review registered only 408 cases between 1888 and 2021 [1]. Although rare, its occurrence is facilitated by several anatomical or connective tissue abnormalities, including polysplenia syndrome, wandering spleen, congenital abdominal anomalies, and conditions associated with ligamentous laxity such as pregnancy, the postpartum period, Marfan syndrome, Down syndrome, and Prader-Willi syndrome [1].

Polysplenia syndrome is a rare congenital syndrome with an estimated incidence of one in 250,000 live births [2,3]. It is a complex condition without pathognomonic features and is associated with various malformations, including pulmonary abnormalities, heterotaxy, and cardiovascular defects [4,5]. Splenic morphology varies, typically presenting as multiple small splenic nodules along the greater curvature of the stomach or as a multilobulated spleen with accessory splenic tissue [6,7]. Patients with polysplenia are predisposed to torsion due to a long and narrow splenic vascular pedicle [8]. Adult presentation is uncommon, as most affected individuals die in childhood from cardiac anomalies; in adults, the diagnosis is often incidental [7,9].

Wandering spleen results from partial or complete absence of the gastrosplenic and splenorenal ligaments, which normally secure the spleen within the left upper quadrant. This abnormal mobility markedly increases the risk of torsion [3]. Its incidence is estimated at 0.2% [10], and it is generally not associated with heterotaxy syndromes.

Situs inversus is a type of heterotaxy syndrome characterized by mirror-image arrangement of thoracic and abdominal organs, with an incidence of one in 10,000 live births [6,11]. It is associated with polysplenia syndrome in 20% of the cases [4,12,13]. However, the coexistence of situs inversus, isolated polysplenia, and wandering spleen within the same individual is exceedingly rare.

We describe a case of splenic torsion in a young adult with situs inversus totalis and concomitant isolated polysplenia and wandering spleen, without additional cardiothoracic or digestive anomalies. To our knowledge, only three similar cases have been reported in the English-language literature [6,8,14]. Although rare, prompt recognition of this condition is crucial, as delayed or incorrect diagnosis may lead to serious complications related to splenic torsion.

## Case presentation

An 18-year-old woman known for situs inversus totalis presented to the emergency department of a regional hospital in Switzerland with a 24-hour history of sudden-onset right-sided abdominal pain associated with vomiting. She reported no bowel disturbances and no fever. Clinical examination revealed localized tenderness in the right upper and lower quadrants without signs of peritonism.

**Figure 1:**
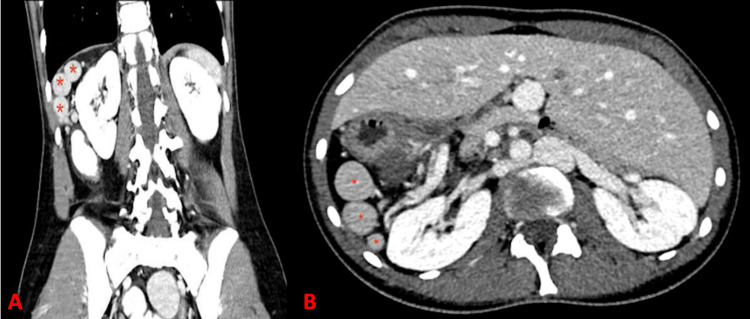
Abdominal CT scan at the time of admission shows multiple accessory spleens (red stars) in the coronal (A) and axial (B) views.

**Figure 2:**
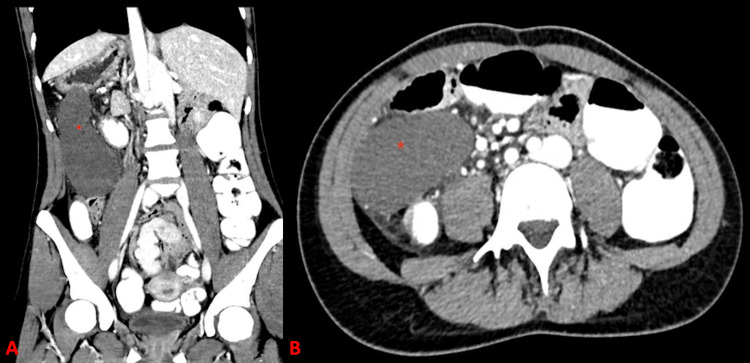
Abdominal CT at the time of admission shows the main spleen with lack of contrast suspected of ischemia (red star) in the coronal (A) and axial (B) views.

Laboratory tests demonstrated leukocytosis (12 G/L; normal range 4-10 G/L) and an elevated C-reactive protein (35 mg/L; normal <10 mg/L). Abdominal CT confirmed situs inversus totalis and revealed polysplenia (Figure 1). A well-demarcated hypodense lesion was identified, consistent with infarction of the main spleen due to torsion of its vascular pedicle (Figure 2). No cardiovascular anomalies were detected.

After urgent transfer to our tertiary care center (Lausanne University Hospital, Lausanne, CHE), a hand-assisted laparoscopic splenectomy was performed. This approach was selected by the operating surgeon to facilitate secure manipulation and extraction of the spleen. The patient was positioned in the left lateral decubitus position. A Pfannenstiel incision was first made for hand-assisted access. Two 12 mm ports were inserted in the right lower and upper quadrants.

**Figure 3:**
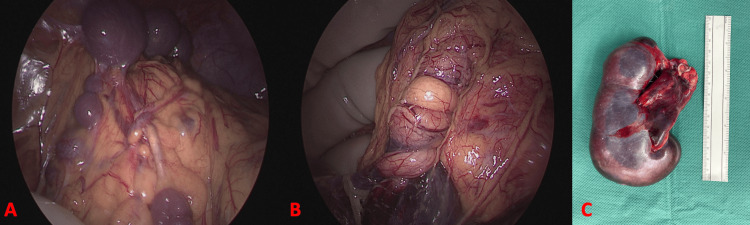
Intraoperative findings and resected spleen submitted for pathological analysis A: Intraoperative view showing multiple accessory spleens, B: Intraoperative view showing torsion of the main splenic pedicle, C: Resected spleen The resected spleen weighed 173 g and measured 11 x 6 x 5.5 cm. The splenic parenchyma had vascular congestion of the red pulp and an area of subcapsular splenic infarction (2 cm long). A congestive splenic hilum lymph node was observed. There were no signs of malignancy.

Exploratory laparoscopy revealed multiple small accessory spleens measuring 0.5 cm to 2 cm (Figure 3, panel A), as well as a larger 5 cm accessory spleen in the right upper quadrant. The main spleen, measuring approximately 10 cm and located in the same region, exhibited a necrotic appearance. The splenic pedicle was twisted, easily palpable, and non-pulsatile (Figure 3, panel B). No reperfusion occurred after detorsion, confirming irreversible splenic infarction. The spleen was otherwise completely mobile within the peritoneal cavity, with no ligamentous attachments. The small accessory spleens located beneath the right diaphragmatic dome were preserved to maintain splenic function and reduce the risk of post-splenectomy infectious complications. Pathological examination showed no evidence of malignancy in the resected spleen (Figure 3, panel C).

Postoperative recovery was uneventful, and the patient was discharged on postoperative day three. The one-month follow-up with her general practitioner revealed mild thrombocytosis (465 G/L; normal range 150-350 G/L), compared with a preoperative level of 301 G/L. No specific treatment or prophylaxis was initiated, and no infectious complications were reported during the 12 months of follow-up.

## Discussion

While our patient was only known to have situs inversus totalis, we intraoperatively diagnosed not only polysplenia with torsion of the main splenic pedicle but also a wandering spleen. Notably, she presented with isolated polysplenia without cardiovascular, pulmonary, or intestinal malformations.

Patients with polysplenia syndrome are predisposed to splenic torsion due to the presence of multiple smaller spleens supplied by narrow pedicles [8]. The first case of splenic torsion in the setting of polysplenia syndrome was reported by Ackermann et al. in 1982 [15], and only 11 additional cases have since been described in the English literature. The coexistence of both a wandering spleen and polysplenia syndrome is exceedingly rare and has been documented only twice [3,16].

Clinical manifestations of splenic torsion are typically nonspecific and commonly include abdominal pain (93%), often associated with nausea and vomiting [1,3]. In cases of torsion-detorsion syndrome (24%), recurrent abdominal pain may lead to misdiagnosis with more frequent conditions. On physical examination, a palpable abdominal mass is present in 76% of patients, while hemodynamic shock is a rare initial presentation [1]. Our patient experienced recurrent right upper quadrant pain for several years, attributed to chronic constipation. These nonspecific symptoms highlight the diagnostic challenge of splenic torsion and underscore the need for clinicians to consider this entity, particularly in individuals with polysplenia syndrome or, as in our case, situs inversus totalis. According to a recent systematic review, only 45% of cases are diagnosed preoperatively [1].

Surgery is the standard of care for splenic torsion. In 2022, Bough et al. [1] published a systematic review including 406 patients, of whom 94% underwent operative management, with 82% treated as emergencies. Splenectomy was the most frequently performed procedure (82%), followed by splenopexy (11%), detorsion alone (1%), and autotransplantation (3%). Among patients initially treated with splenopexy, 6% required secondary splenectomy, whereas 52% of those managed conservatively required subsequent surgery. Laparoscopic management has been increasingly reported and has demonstrated comparable outcomes to open surgery, with the exception of a higher rate of prolonged postoperative ileus [1].

The spleen plays a key role in host immunity. Patients with heterotaxy syndrome and asplenia have no splenic function and are thus at high risk of severe bacterial infections [17,18]. Evaluation of hyposplenism includes detection of Howell-Jolly bodies, pitted red cells, and circulating IgM memory B cells [18]. However, splenic function in polysplenia remains poorly understood, and no formal guidelines exist regarding its evaluation.

An Italian study from 2016 showed that children with heterotaxy syndrome and asplenia had a significantly higher risk of severe infections compared with patients with heterotaxy syndrome and a normal spleen or polysplenia [19]. Conversely, Loomba et al. [20] concluded that heterotaxy syndrome itself confers a high risk of bacteremia, irrespective of whether patients have asplenia, polysplenia, or a normally formed spleen. As splenic function is not solely determined by the presence or location of splenic tissue, the authors advocated for managing all heterotaxy patients according to guidelines for hyposplenism, even when splenic anatomy appears preserved.

Our patient had no known polysplenia before surgery. Given the absence of clinical evidence of hyposplenism, it is reasonable to assume that her splenic function was preserved prior to the splenectomy, although specific tests were not performed to confirm this. Her 12-month postoperative course was uneventful.

## Conclusions

Splenic torsion is a rare but serious condition that requires prompt diagnosis and surgical management. Patients with situs inversus totalis, polysplenia, and wandering spleen are predisposed to this complication due to their abnormal anatomy. Imaging via CT is essential for identifying splenic torsion and assessing splenic viability. When an infarction is present, splenectomy remains the gold standard, and laparoscopic splenectomy represents a safe and effective approach even in complex anatomical situations.
